# Editorial: Radiotherapy strategies for precise treatment on brain metastases

**DOI:** 10.3389/fonc.2024.1366261

**Published:** 2024-03-20

**Authors:** Eric J. Lehrer, William G. Breen, Ugur Sener, Jian L. Campian

**Affiliations:** ^1^ Department of Radiation Oncology, Mayo Clinic, Rochester, MN, United States; ^2^ Department of Neurology, Mayo Clinic, Rochester, MN, United States; ^3^ Department of Medical Oncology, Mayo Clinic, Rochester, MN, United States

**Keywords:** brain metastases (BM), immunotherapy, radiation oncology, neuro oncology, neurosurgery, radiation necrosis (RN)

Brain metastases (BrM) are the most common intracranial neoplasm in adults, with an estimated annual incidence between 8-10% (1–3). The incidence of BrM is expected to increase due to advancements in systemic therapies, which have improved overall survival (OS) and progression-free survival (PFS) in multiple advanced solid malignancies (4–7). Multidisciplinary management of BrM is essential with participation from neurosurgery, radiation oncology, medical oncology, and neurology. In the past three decades there have been marked advancements in surgical techniques, radiation therapy, systemic therapies, and supportive care which have improved outcomes and tolerability of treatment in patients with BrM (8).

Surgical resection is typically reserved for patients who have large symptomatic tumors with associated mass effect in an accessible location (9). Resection or biopsy is also considered to establish tissue diagnosis. Due to its association with tumor control and decreased risk of neurologic death, adjuvant whole brain radiotherapy (WBRT) has traditionally been recommended after resection (10). However, conventional WBRT is associated with an increased risk of neurocognitive side effects, which can significantly impair quality of life (11–14). Therefore, techniques that have increased dose conformality, such as stereotactic radiosurgery (SRS) have become more commonly utilized in the management of BrM (11, 13, 15). Furthermore, interstitial radiation techniques, (e.g., intraoperative radiotherapy and brachytherapy), as well as heavy ion therapy (e.g., carbon ion therapy) are presently being explored (16, 17). Systemic therapies such as chemotherapy, immunotherapy, and targeted agents can be administered alone or in combination in certain settings, with increasing rates of intracranial response (18, 19). Treatment management decisions in patients with BrM requires consideration of multiple patient-specific factors, such as performance status, tumor size and location, number of metastases, tumor histology, driver mutations, and availability of CNS-penetrant systemic therapy (8). While CNS-penetrant therapies are a promising treatment strategy, exclusively utilizing these therapies without radiotherapy is a strategy in its relative infancy and requires further investigation (9). Additionally, an important multiinstitutional retrospective analysis suggested that patient outcomes were improved when patients who were eligible for EGFR-TKI agents underwent SRS first followed by EGFR-TKI (versus those who deferred radiotherapy) (20). This Research Topic of *Frontiers in Oncology* explores different approaches in personalizing treatment in patients with brain metastases.

Deng et al. conducted a propensity score matched study of 291 patients with EGFR-mutant non-small cell lung cancer (NSCLC) BrM. Patients received EGFR-TKI alone or EGFR-TKI with cranial radiotherapy. A highly heterogeneous radiotherapy dosing paradigm was used with most patients receiving WBRT with or without the incorporation of a boost. The authors observed statistically significant improvements in both intracranial PFS (14.7 months versus 8.9 months) and OS (45.3 months versus 32.1 months). These findings do support the need for further personalization of care in the management of BrM. However, further validation is needed, such as verification in the randomized setting, reporting of detailed toxicity data, and utilization of more uniform radiotherapy dosing and techniques (e.g., SRS).

The role of prognostic scoring systems to inform treatment decisions in BrM management have been utilized for decades (8, 21). Specifically, the latest edition of the diagnosis-specific graded prognostic assessment (DS-GPA) has incorporated molecular markers into its scoring system (8). Nieder et al. conducted a retrospective study of 198 patients with BrM who underwent WBRT, aimed at expanding upon the laboratory parameters in patients with brain metastases (LabBM) score. The auhors findings suggest that the LabBM score may be further refined to aid in prognostication of patients with BrM undergoing WBRT. Li et al. conducted a retrospective study of 116 patients with colorectal BrM who underwent SRS or stereotactic radiotherapy. Colorectal BrM are associated with a poor prognosis and their intrinsic radioresistance presents multiple treatment challenges (8). In addition to a low DS-GPA score and the presence of extracranial disease, the authors observed that the presence of a KRAS mutation was associated with decreased OS. While this study is retrospective in nature, these findings do suggest that the incorporation of KRAS mutation status may be considered in future BrM prognostic scoring systems.

Kong et al. conducted a study of 76 patients with NSCLC who experienced progression of a pre-existing or development of a new BrM after at least one line of prior systemic therapy. All patients received WBRT with or without the addition of concurrent and maintenance anlotinib. Anlotinib, a potent inhibitor of VEGF has been studied in the phase 3 setting with data suggesting improved intracranial disease control while also demonstrating an association with increased neurotoxicity and psychosocial symptoms (22). The authors did observe a mild intracranial PFS improvement with anlotinib (6.7 months versus 5.3 months; *p* = 0.04). Grade 3-5 toxicities were observed in approximately 15% of patients in the anlotinib group, including one fatal pulmonary hemorrhage. Thus, further investigation is warranted.

While WBRT is associated with a higher risk of cognitive sequelae compared to SRS, not all patients are candidates for SRS. This can be due to multiple factors, such as the overall burden of intracranial disease or lack of availability of radiosurgical platforms and the requisite expertise to deliver SRS. In recent years, the incorporation of memantine and/or hippocampal avoidance into WBRT have allowed for improvements in cognitive outcomes (12, 14). HA-WBRT utilizes advanced and modern radiotherapeutic approaches, which requires a high level of expertise from physicists, clinicians, and dosimetrists. Xue et al. presented a study that utilized a simplified non-coplanar volumetric arc therapy approach for HA-WBRT planning. They observed that this approach has the potential to further reduce dose to the hippocampus and other critical intracranial structures. While this does require further investigation, this may prove to be a strategy to miminize treatment-related sequelae in these patients.

HA-WBRT is also being utilized in the setting of prophylactic cranial irradiation (PCI) in select patients with small cell lung cancer (SCLC). Wang et al. performed a meta-analysis of 15 studies of patients with extensive stage SCLC (ES-SCLC) who underwent PCI. While a benefit in OS was not observed, a lower incidence of BrM development was noted. These data support the landmark findings by Takahashi et al., which support close observation over PCI in these patients (23).

Radiation treatment is a major component of the BrM armamentarium. In recent years, advancements in treatment delivery, patient selection methodologies, identification of molecular mutations, and systemic therapies have allowed for more precise and tailored treatments for these patients. Ongoing clinical trials will enhance the personalization of radiotherapy delivery in BrM patients.

## Author contributions

EL: Conceptualization, Writing – original draft, Writing – review & editing. WB: Writing – original draft, Writing – review & editing. US: Writing – original draft, Writing – review & editing. JC: Project administration, Supervision, Writing – original draft, Writing – review & editing.
